# The genome sequence of the crisped pincushion, *Ulota crispa* (Hedw.) Brid.

**DOI:** 10.12688/wellcomeopenres.23429.1

**Published:** 2024-12-03

**Authors:** David Bell, Royal Botanic Garden Edinburgh Genome Acquisition Lab, Darwin Tree of Life Barcoding collective, Wellcome Sanger Institute Tree of Life Management, Samples and Laboratory team, Wellcome Sanger Institute Scientific Operations: Sequencing Operations, Wellcome Sanger Institute Tree of Life Core Informatics team, Tree of Life Core Informatics collective, Darwin Tree of Life Consortium

**Affiliations:** 1 Royal Botanic Garden Edinburgh, Edinburgh, Scotland, UK

**Keywords:** Ulota crispa, crisped pincushion, genome sequence, chromosomal, Orthotrichales

## Abstract

We present a genome assembly from a haploid *Ulota crispa* gametophyte (the crisped pincushion; Streptophyta; Bryopsida; Orthotrichales; Orthotrichaceae). The genome sequence spans 275.00 megabases. Most of the assembly is scaffolded into 11 chromosomal pseudomolecules. The mitochondrial and plastid genome assemblies have lengths of 104.64 kilobases and 123.54 kilobases, respectively.

## Species taxonomy

Eukaryota; Viridiplantae; Streptophyta; Streptophytina; Embryophyta; Bryophyta; Bryophytina; Bryopsida; Bryidae; Bryanae; Orthotrichales; Orthotrichaceae; *Ulota*; *Ulota crispa* (Hedw.) Brid. (NCBI:txid140636).

## Background

*Ulota crispa* (Hedw.) Brid., also known as crisped pincushion, is an epiphytic moss which grows as tufts on twigs and branches, particularly in wet woodland. It is common and widespread in Europe, and also known from east Asia (China, Russian Far East and Japan, and Taiwan) and western North America, where it is uncommon ( Caparrós *et al.*, 2016). It is widespread in Britain and Ireland ( Blockeel, 2017).

Caparrós *et al.* (2016) used morphological and molecular data to demonstrate that the *Ulota crispa* complex consisted of three distinct taxa, reinstating *Ulota crispula* Bruch and *U. intermedia* Schimp. at the species level, as distinct from *Ulota crispa* s. str.

*Ulota crispa* is a monoicous species, producing male and female reproductive structures on the same plant. Capsules are common, maturing in summer ( Blockeel, 2017).

The chromosomally complete genome presented here is consistent with chromosome counts reported for Britain by Smith & Newton (1967). Other chromosome counts reported from Irish ( Vaarama, 1956) and Scottish ( Ramsay, 1969) populations suggest additional variability, but due to the past taxonomic confusion it is unclear which counts are attributable to *U. crispa* s. str. and which may represent other species in the complex. The genome of *Ulota crispa* was sequenced as part of the Darwin Tree of Life Project, a collaborative effort to sequence all named eukaryotic species in the Atlantic Archipelago of Britain and Ireland. We anticipate this high-quality reference genome will be a valuable genomic resource for a range of future studies.

## Genome sequence report

**Figure 1.**
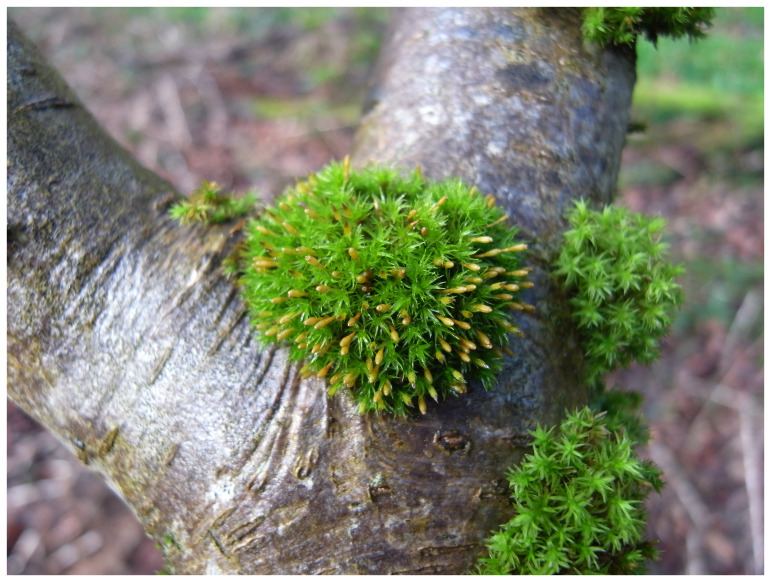
Photograph of the *Ulota crispa* population from which samples used for genome sequencing were taken.

The genome of a *Ulota crispa* gamtophyte ( Figure 1) was sequenced using Pacific Biosciences single-molecule HiFi long reads, generating a total of 21.28 Gb (gigabases) from 2.18 million reads, providing approximately 117-fold coverage. Primary assembly contigs were scaffolded with chromosome conformation Hi-C data, which produced 101.87 Gb from 674.62 million reads. Specimen and sequencing details are provided in Table 1.

**Table 1.** Specimen and sequencing data for *Ulota crispa*.

Project information			
**Study title**	Ulota crispa (crisped pincushion)		
**Umbrella BioProject**	PRJEB70976		
**Species**	*Ulota crispa*		
**BioSample**	SAMEA14392285		
**NCBI taxonomy ID**	140636		
Specimen information			
**Technology**	**ToLID**	**BioSample ** **accession**	**Organism ** **part**
**PacBio long read sequencing**	cbUloCris4	SAMEA14392356	shoot
**Hi-C sequencing**	cbUloCris1	SAMEA14392353	shoot
Sequencing information			
**Platform**	**Run accession**	**Read count**	**Base count (Gb)**
**Hi-C Illumina NovaSeq 6000**	ERR12356314	6.75e+08	101.87
**PacBio Sequel IIe**	ERR12370309	2.18e+06	21.28

**Figure 2.**
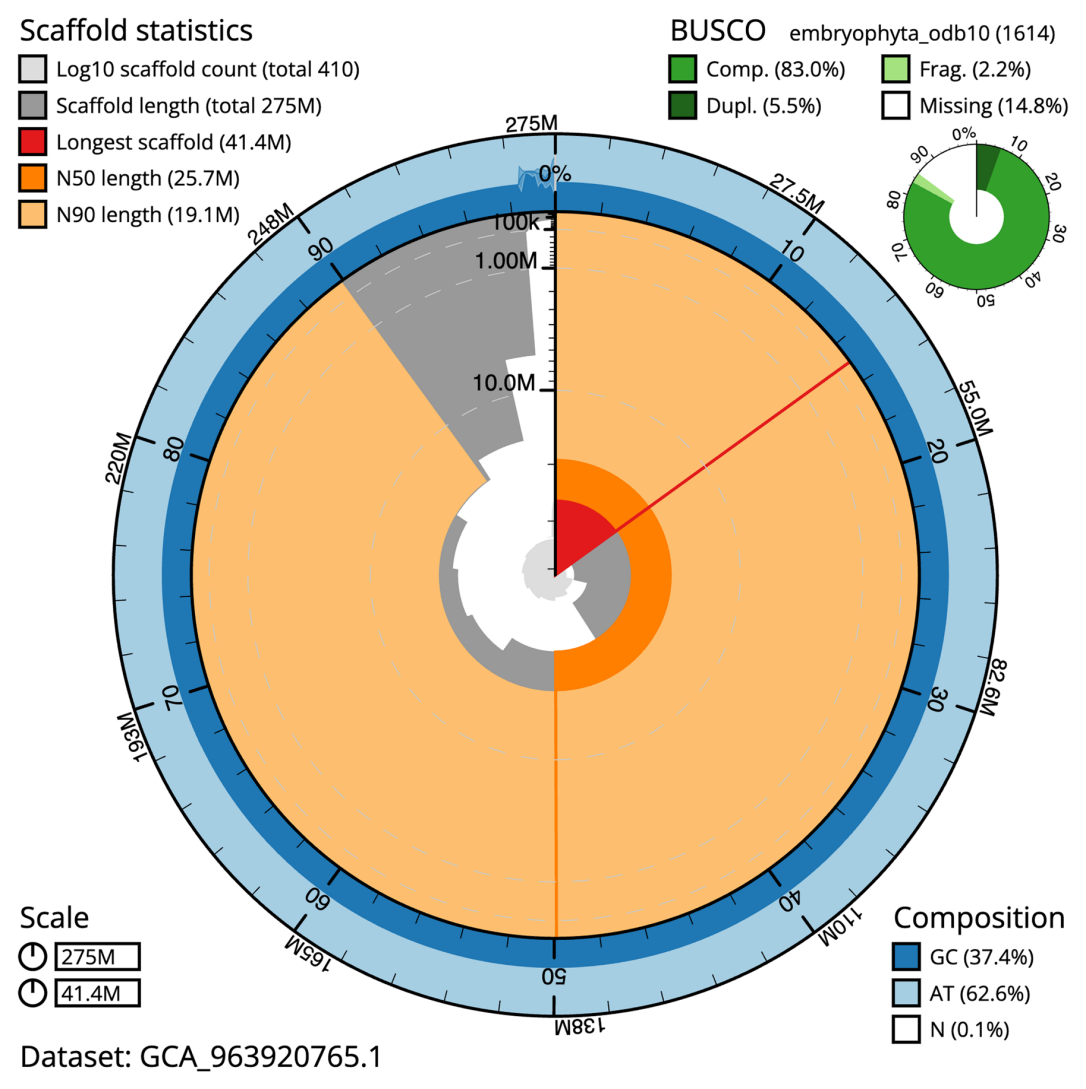
Genome assembly of *Ulota crispa*, cbUloCris4.1: metrics. The BlobToolKit snail plot shows N50 metrics and BUSCO gene completeness. The main plot is divided into 1,000 size-ordered bins around the circumference with each bin representing 0.1% of the 275,217,579 bp assembly. The distribution of scaffold lengths is shown in dark grey with the plot radius scaled to the longest scaffold present in the assembly (41,373,197 bp, shown in red). Orange and pale-orange arcs show the N50 and N90 scaffold lengths (25,686,176 and 19,138,555 bp), respectively. The pale grey spiral shows the cumulative scaffold count on a log scale with white scale lines showing successive orders of magnitude. The blue and pale-blue area around the outside of the plot shows the distribution of GC, AT and N percentages in the same bins as the inner plot. A summary of complete, fragmented, duplicated and missing BUSCO genes in the embryophyta_odb10 set is shown in the top right. An interactive version of this figure is available at https://blobtoolkit.genomehubs.org/view/Ulota_crispa/dataset/GCA_963920765.1/snail.

**Figure 3.**
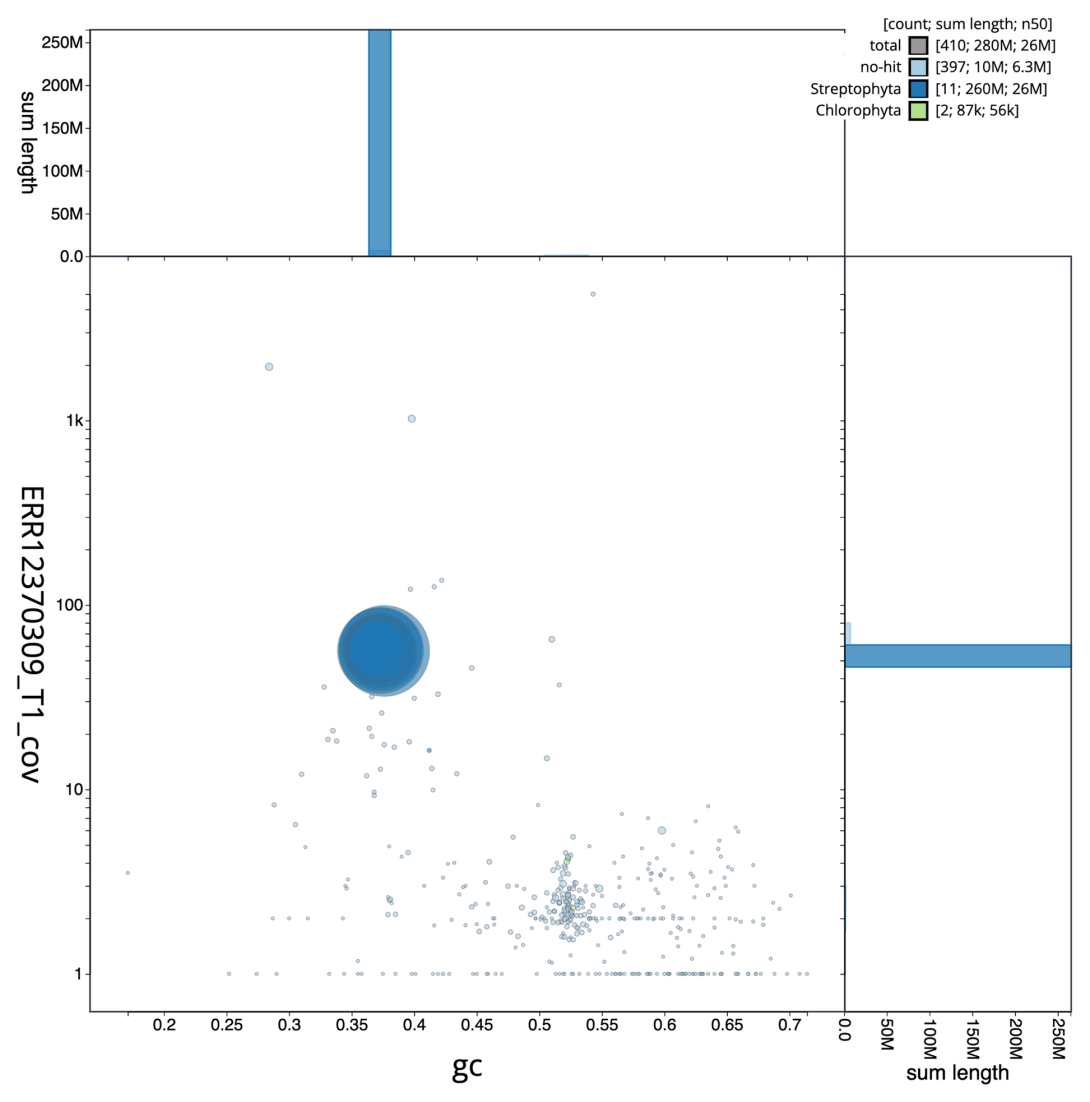
Genome assembly of *Ulota crispa*, cbUloCris4.1: BlobToolKit GC-coverage plot. Scaffolds are coloured by phylum. Circles are sized in proportion to scaffold length. Histograms show the distribution of scaffold length sum along each axis. An interactive version of this figure is available at https://blobtoolkit.genomehubs.org/view/Ulota_crispa/dataset/GCA_963920765.1/blob.

**Figure 4.**
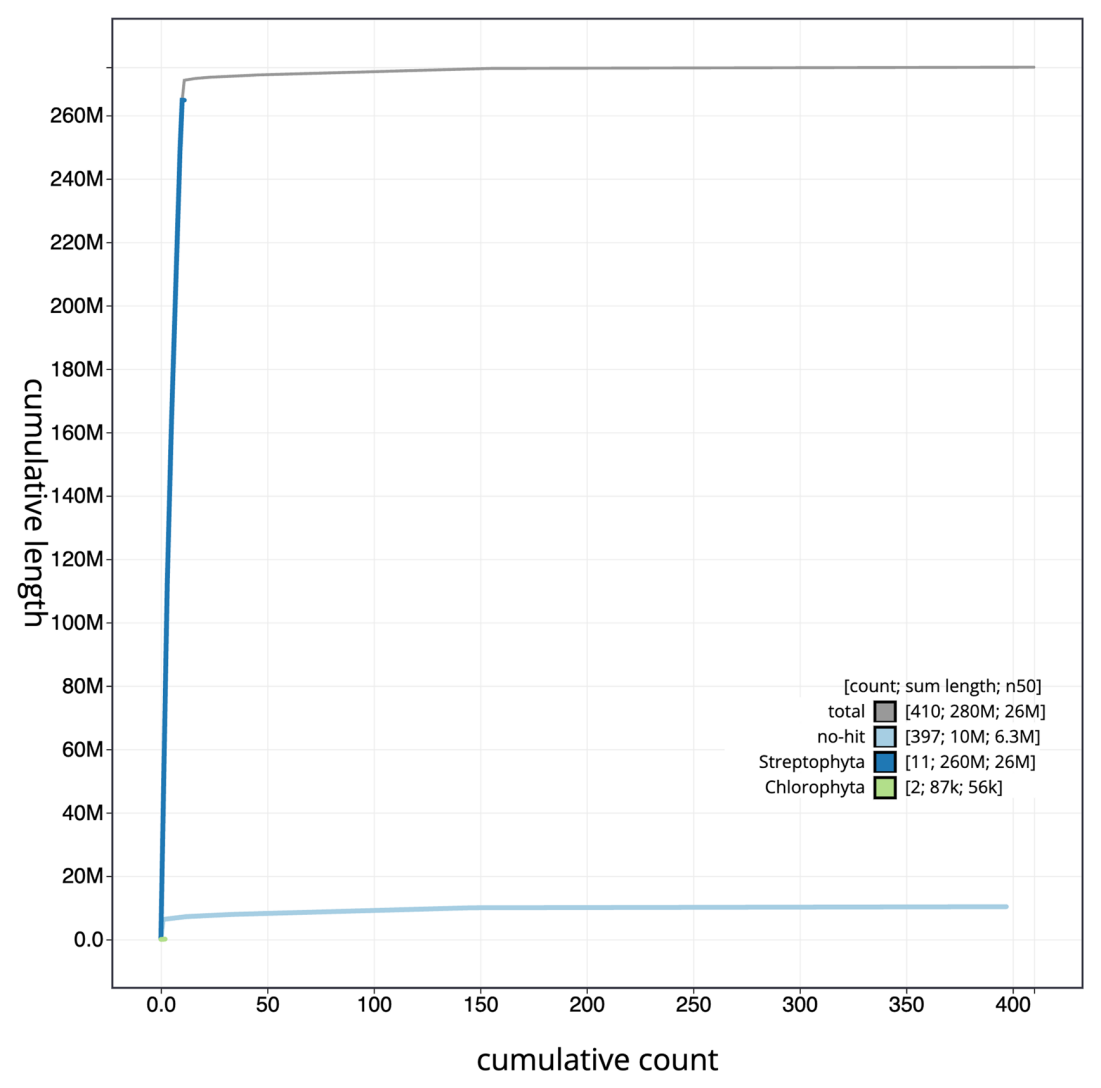
Genome assembly of *Ulota crispa*, cbUloCris4.1: BlobToolKit cumulative sequence plot. The grey line shows cumulative length for all scaffolds. Coloured lines show cumulative lengths of scaffolds assigned to each phylum using the buscogenes taxrule. An interactive version of this figure is available at https://blobtoolkit.genomehubs.org/view/Ulota_crispa/dataset/GCA_963920765.1/cumulative.

**Figure 5.**
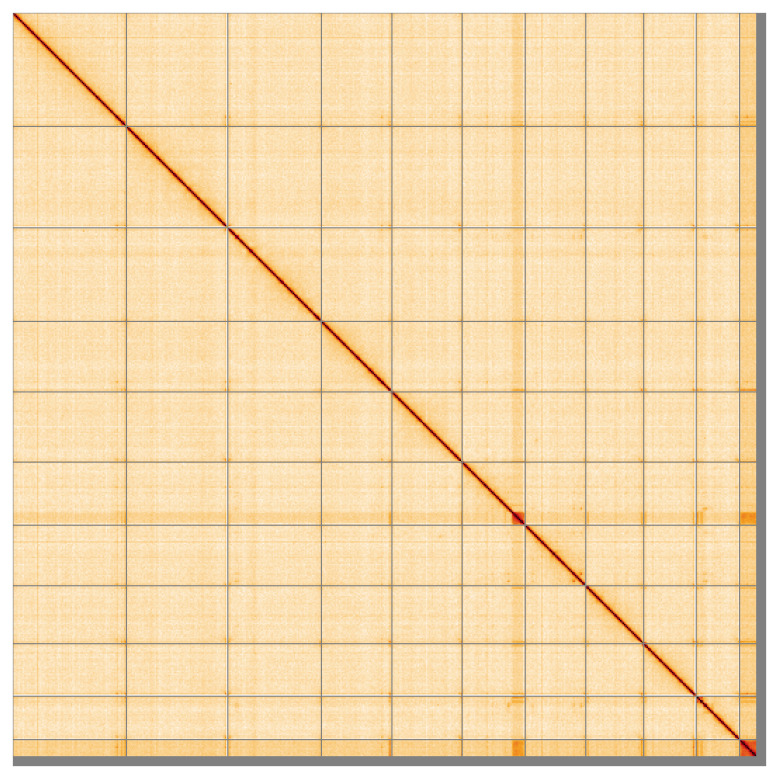
Genome assembly of *Ulota crispa*, cbUloCris4.1: Hi-C contact map of the cbUloCris4.1 assembly, visualised using HiGlass. Chromosomes are shown in order of size from left to right and top to bottom. An interactive version of this figure may be viewed at https://genome-note-higlass.tol.sanger.ac.uk/l/?d=HayxlpI1Qt-Js93QnMSdkw.

Manual assembly curation corrected 50 missing joins or mis-joins, reducing the assembly length by 3.65%, and decreasing the scaffold N50 by 24.3%. The final assembly has a total length of 275.00 Mb in 408 sequence scaffolds with a scaffold N50 of 25.7 Mb ( Table 2), and a total gap count of 742. The snail plot in Figure 2 provides a summary of the assembly statistics, while the distribution of assembly scaffolds on GC proportion and coverage is shown in Figure 3. The cumulative assembly plot in Figure 4 shows curves for subsets of scaffolds assigned to different phyla. Most (98.5%) of the assembly sequence was assigned to 11 chromosomal-level scaffolds. Chromosome-scale scaffolds confirmed by the Hi-C data are named in order of size ( Figure 5; Table 3). While not fully phased, the assembly deposited is of one haplotype. Contigs corresponding to the second haplotype have also been deposited. The mitochondrial and plastid genomes were also assembled and can be found as contigs within the multifasta file of the genome submission.

**Table 2.** Genome assembly data for *Ulota crispa*, cbUloCris4.1.

Genome assembly		
Assembly name	cbUloCris4.1	
Assembly accession	GCA_963920765.1	
Span (Mb)	275.00	
Number of contigs	1,152	
Number of scaffolds	408	
Longest scaffold (Mb)	41.37	
Assembly metrics *		*Benchmark*
Contig N50 length (Mb)	0.6	*≥ 1 Mb*
Scaffold N50 length (Mb)	25.7	*= chromosome N50*
Consensus quality (QV)	60.9	*≥ 40*
*k*-mer completeness	100.0%	*≥ 95%*
BUSCO v5.4.3 lineage: embryophyta_odb10	C:82.6%[S:77.3%,D:5.3%], F:2.5%,M:14.9%,n:1,614	*S > 90%, D < 5%*
Percentage of assembly mapped to chromosomes	98.5%	*≥ 90%*
Organelles	Mitochondrial genome: 104.64 kb Plastid genome: 123.54 kb	*complete single alleles*

* Assembly metric benchmarks are adapted from Rhie *et al.* (2021) and the Earth BioGenome Project Report on Assembly Standards September 2024. ** BUSCO scores based on the embryophyta_odb10 BUSCO set using version 5.4.3. C = complete [S = single copy, D = duplicated], F = fragmented, M = missing, n = number of orthologues in comparison. A full set of BUSCO scores is available at https://blobtoolkit.genomehubs.org/view/Ulota_crispa/dataset/GCA_963920765.1/busco.

**Table 3.** Chromosomal pseudomolecules in the genome assembly of *Ulota crispa*, cbUloCris4.

INSDC accession	Name	Length (Mb)	GC%
OY987242.1	1	41.37	37.5
OY987243.1	2	36.98	37.5
OY987244.1	3	34.07	37.5
OY987245.1	4	25.69	37.0
OY987246.1	5	25.64	37.5
OY987247.1	6	22.92	37.0
OY987248.1	7	22.04	37.0
OY987249.1	8	21.16	37.5
OY987250.1	9	19.14	37.0
OY987251.1	10	15.79	37.0
OY987252.1	11	6.3	37.0
OY987253.1	MT	0.1	40.0
OY987254.1	Pltd	0.12	28.5

The estimated Quality Value (QV) of the final assembly is 60.9 with *k*-mer completeness of 100.0%, and the assembly has a BUSCO v completeness of 82.6% (single = 77.3%, duplicated = 5.3%), using the embryophyta_odb10 reference set ( *n* = 1,614).

Metadata for specimens, BOLD barcode results, spectra estimates, sequencing runs, contaminants and pre-curation assembly statistics are given at https://links.tol.sanger.ac.uk/species/140636.

## Methods

### Sample acquisition and DNA barcoding

*Ulota crispa* specimens were collected from General Monck’s Battery, Roslin Glen Country Park, Midlothian, Scotland, UK (latitude 55.86, longitude –3.16) on 2022-02-21. The specimens were collected and identified by David Bell (Royal Botanic Garden Edinburgh) and preserved by flash freezing in liquid nitrogen. One gametophyte (specimen ID EDTOL03207, ToLID cbUloCris4) was used for Hi-Fi DNA sequencing and another (specimen ID EDTOL03204, ToLID cbUloCris1) was used for Hi-C sequencing. The herbarium specimen associated with the sequenced plant is kept at the Royal Botanic Garden Edinburgh (E) https://data.rbge.org.uk/herb/E01152200.

The initial species identification was verified by an additional DNA barcoding process following the framework developed by Twyford *et al.* (2024). Part of the plant specimen was preserved in silica gel desiccant ( Chase & Hills, 1991). DNA was extracted from the dried specimen, then PCR was used to amplify standard barcode regions. The resulting amplicons were sequenced and compared to public sequence databases including GenBank and the Barcode of Life Database (BOLD). The barcode sequences for this specimen are available on BOLD ( Ratnasingham & Hebert, 2007). Following whole genome sequence generation, DNA barcodes were also used alongside the initial barcoding data for sample tracking through the genome production pipeline at the Wellcome Sanger Institute ( Twyford *et al.*, 2024). The standard operating procedures for the Darwin Tree of Life barcoding have been deposited on protocols.io ( Beasley *et al.*, 2023).

### Nucleic acid extraction

The workflow for high molecular weight (HMW) DNA extraction at the Wellcome Sanger Institute (WSI) Tree of Life Core Laboratory includes a sequence of procedures: sample preparation and homogenisation, DNA extraction, fragmentation and purification. Detailed protocols are available on protocols.io ( Denton *et al.*, 2023b). The cbUloCris4 sample was weighed and dissected on dry ice ( Jay *et al.*, 2023). Tissue from a shoot of the specimen was homogenised using a PowerMasher II tissue disruptor ( Denton *et al.*, 2023a). HMW DNA was extracted using the Automated Plant MagAttract v4 protocol ( Jackson & Howard, 2023). HMW DNA was sheared into an average fragment size of 12–20 kb in a Megaruptor 3 system ( Bates *et al.*, 2023). Sheared DNA was purified by solid-phase reversible immobilisation, using AMPure PB beads to eliminate shorter fragments and concentrate the DNA ( Oatley *et al.*, 2023). The concentration of the sheared and purified DNA was assessed using a Nanodrop spectrophotometer, Qubit Fluorometer and Qubit dsDNA High Sensitivity Assay kit. Fragment size distribution was evaluated by running the sample on the FemtoPulse system.

### Hi-C preparation

Hi-C data were generated from the sample at the WSI Scientific Operations core, using the Arima-HiC v2 kit. Tissue was finely ground using cryoPREP, and then subjected to nuclei isolation using a modified protocol of the Qiagen QProteome Kit. After isolation, the nuclei were fixed, and the DNA crosslinked using a 37% formaldehyde solution. The crosslinked DNA was then digested using the restriction enzyme master mix. The 5’-overhangs were then filled in and labelled with biotinylated nucleotides and proximally ligated. An overnight incubation was carried out for enzymes to digest remaining proteins and for crosslinks to reverse. A clean up was performed with SPRIselect beads prior to library preparation. DNA concentration was quantified using the Qubit Fluorometer v2.0 and Qubit HS Assay Kit according to the manufacturer’s instructions.

### Library preparation and sequencing

Library preparation and sequencing were performed at the WSI Scientific Operations core. Pacific Biosciences HiFi circular consensus DNA sequencing libraries were prepared using the PacBio Express Template Preparation Kit v2.0 (Pacific Biosciences, California, USA) as per the manufacturer's instructions. The kit includes the reagents required for removal of single-strand overhangs, DNA damage repair, end repair/A-tailing, adapter ligation, and nuclease treatment. Library preparation also included a library purification step using AMPure PB beads (Pacific Biosciences, California, USA) and size selection step to remove templates shorter than 3 kb using AMPure PB modified SPRI. DNA concentration was quantified using the Qubit Fluorometer v2.0 and Qubit HS Assay Kit and the final library fragment size analysis was carried out using the Agilent Femto Pulse Automated Pulsed Field CE Instrument and gDNA 165kb gDNA and 55kb BAC analysis kit. Samples were sequenced using the Sequel IIe system (Pacific Biosciences, California, USA). The concentration of the library loaded onto the Sequel IIe was in the range 40–135 pM. The SMRT link software, a PacBio web-based end-to-end workflow manager, was used to set-up and monitor the run, as well as perform primary and secondary analysis of the data upon completion.

For Hi-C library preparation, DNA was fragmented to a size of 400 to 600 bp using a Covaris E220 sonicator. The DNA was then enriched, barcoded, and amplified using the NEBNext Ultra II DNA Library Prep Kit following manufacturers’ instructions. The Hi-C sequencing was performed using paired-end sequencing with a read length of 150 bp on an Illumina NovaSeq 6000 instrument.

### Genome assembly, curation and evaluation

***Assembly***

The HiFi reads were first assembled using Hifiasm ( Cheng *et al.*, 2021) with the --primary option. The Hi-C reads were mapped to the primary contigs using bwa-mem2 ( Vasimuddin *et al.*, 2019). The contigs were further scaffolded using the provided Hi-C data ( Rao *et al.*, 2014) in YaHS ( Zhou *et al.*, 2023) using the --break option for handling potential misassemblies. The scaffolded assemblies were evaluated using Gfastats ( Formenti *et al.*, 2022), BUSCO ( Manni *et al.*, 2021) and MERQURY.FK ( Rhie *et al.*, 2020). The organelle genomes were assembled using OATK ( Zhou *et al.*, 2024).

***Curation***

The assembly was checked for contamination using and corrected using the TreeVal pipeline ( Pointon *et al.*, 2023). Manual curation was performed using JBrowse2 ( Diesh *et al.*, 2023), HiGlass ( Kerpedjiev *et al.*, 2018) and PretextView ( Harry, 2022). The workflow and documentation for rapid curation are provided at https://gitlab.com/wtsi-grit/rapid-curation.

***Evaluation of final assembly***

The final assembly was post-processed and evaluated using the three Nextflow ( Di Tommaso *et al.*, 2017) DSL2 pipelines: sanger-tol/readmapping ( Surana *et al.*, 2023a), sanger-tol/genomenote ( Surana *et al.*, 2023b), and sanger-tol/blobtoolkit ( Muffato *et al.*, 2024). The readmapping pipeline aligns the Hi-C reads using bwa-mem2 ( Vasimuddin *et al.*, 2019) and combines the alignment files with SAMtools ( Danecek *et al.*, 2021). The “genomenote” pipeline converts the Hi-C alignments into a contact map using BEDTools ( Quinlan & Hall, 2010) and the Cooler tool suite ( Abdennur & Mirny, 2020). The contact map is visualised in HiGlass ( Kerpedjiev *et al.*, 2018). This pipeline also computes *k*-mer completeness and QV consensus quality values with FastK and MERQURY.FK, and runs BUSCO ( Manni *et al.*, 2021) to assess completeness.

The blobtoolkit pipeline is a Nextflow port of the previous Snakemake Blobtoolkit pipeline ( Challis *et al.*, 2020). It aligns the PacBio reads in SAMtools and minimap2 ( Li, 2018) and generates coverage tracks for regions of fixed size. In parallel, it queries the GoaT database ( Challis *et al.*, 2023) to identify all matching BUSCO lineages to run BUSCO ( Manni *et al.*, 2021). For the three domain-level BUSCO lineages, the pipeline aligns the BUSCO genes to the UniProt Reference Proteomes database ( Bateman *et al.*, 2023) with DIAMOND blastp ( Buchfink *et al.*, 2021). The genome is also divided into chunks according to the density of the BUSCO genes from the closest taxonomic lineage, and each chunk is aligned to the UniProt Reference Proteomes database using DIAMOND blastx. Genome sequences without a hit are chunked using seqtk and aligned to the NT database with blastn ( Altschul *et al.*, 1990). The blobtools suite combines all these outputs into a blobdir for visualisation.

The genome assembly and evaluation pipelines were developed using nf-core tooling ( Ewels *et al.*, 2020) and MultiQC ( Ewels *et al.*, 2016), relying on the Conda package manager, the Bioconda initiative ( Grüning *et al.*, 2018), the Biocontainers infrastructure ( da Veiga Leprevost *et al.*, 2017), as well as the Docker ( Merkel, 2014) and Singularity ( Kurtzer *et al.*, 2017) containerisation solutions.

Table 4 contains a list of relevant software tool versions and sources.

**Table 4.** Software tools: versions and sources.

Software tool	Version	Source
BEDTools	2.30.0	https://github.com/arq5x/bedtools2
BLAST	2.14.0	ftp://ftp.ncbi.nlm.nih.gov/blast/executables/blast+/
BlobToolKit	4.3.7	https://github.com/blobtoolkit/blobtoolkit
BUSCO	5.4.3 and 5.5.0	https://gitlab.com/ezlab/busco
bwa-mem2	2.2.1	https://github.com/bwa-mem2/bwa-mem2
Cooler	0.8.11	https://github.com/open2c/cooler
DIAMOND	2.1.8	https://github.com/bbuchfink/diamond
fasta_windows	0.2.4	https://github.com/tolkit/fasta_windows
FastK	427104ea91c78c3b8b8b49f1a7d6bbeaa869ba1c	https://github.com/thegenemyers/FASTK
Gfastats	1.3.6	https://github.com/vgl-hub/gfastats
GoaT CLI	0.2.5	https://github.com/genomehubs/goat-cli
Hifiasm	0.19.5-r587	https://github.com/chhylp123/hifiasm
HiGlass	44086069ee7d4d3f6f3f0012569789ec138f42b84a a44357826c0b6753eb28de	https://github.com/higlass/higlass
Merqury.FK	d00d98157618f4e8d1a9190026b19b471055b22e	https://github.com/thegenemyers/MERQURY.FK
MultiQC	1.14, 1.17, and 1.18	https://github.com/MultiQC/MultiQC
NCBI Datasets	15.12.0	https://github.com/ncbi/datasets
Nextflow	23.04.0-5857	https://github.com/nextflow-io/nextflow
PretextView	0.2	https://github.com/sanger-tol/PretextView
OATK	0.9	https://github.com/c-zhou/oatk
samtools	1.16.1, 1.17, and 1.18	https://github.com/samtools/samtools
sanger-tol/ genomeassembly	0.10.0	https://github.com/sanger-tol/genomeassembly
sanger-tol/ genomenote	1.1.1	https://github.com/sanger-tol/genomenote
sanger-tol/ readmapping	1.2.1	https://github.com/sanger-tol/readmapping
Seqtk	1.3	https://github.com/lh3/seqtk
Singularity	3.9.0	https://github.com/sylabs/singularity
TreeVal	1.0.0	https://github.com/sanger-tol/treeval
YaHS	1.2a.2	https://github.com/c-zhou/yahs

### Wellcome Sanger Institute – Legal and Governance

The materials that have contributed to this genome note have been supplied by a Darwin Tree of Life Partner. The submission of materials by a Darwin Tree of Life Partner is subject to the **‘Darwin Tree of Life Project Sampling Code of Practice’**, which can be found in full on the Darwin Tree of Life website here. By agreeing with and signing up to the Sampling Code of Practice, the Darwin Tree of Life Partner agrees they will meet the legal and ethical requirements and standards set out within this document in respect of all samples acquired for, and supplied to, the Darwin Tree of Life Project.

Further, the Wellcome Sanger Institute employs a process whereby due diligence is carried out proportionate to the nature of the materials themselves, and the circumstances under which they have been/are to be collected and provided for use. The purpose of this is to address and mitigate any potential legal and/or ethical implications of receipt and use of the materials as part of the research project, and to ensure that in doing so we align with best practice wherever possible. The overarching areas of consideration are:

•    Ethical review of provenance and sourcing of the material

•    Legality of collection, transfer and use (national and international) 

Each transfer of samples is further undertaken according to a Research Collaboration Agreement or Material Transfer Agreement entered into by the Darwin Tree of Life Partner, Genome Research Limited (operating as the Wellcome Sanger Institute), and in some circumstances other Darwin Tree of Life collaborators.

## Data Availability

European Nucleotide Archive: *Ulota crispa* (crisped pincushion). Accession number PRJEB70976; https://identifiers.org/ena.embl/PRJEB70976. The genome sequence is released openly for reuse. The *Ulota crispa* genome sequencing initiative is part of the Darwin Tree of Life (DToL) project. All raw sequence data and the assembly have been deposited in INSDC databases. The genome will be annotated using available RNA-Seq data and presented through the Ensembl pipeline at the European Bioinformatics Institute. Raw data and assembly accession identifiers are reported in Table 1.

## Author information

Members of the Royal Botanic Garden Edinburgh Genome Acquisition Lab are listed here: https://doi.org/10.5281/zenodo.4786682.

Members of the Darwin Tree of Life Barcoding collective are listed here: https://doi.org/10.5281/zenodo.4893703.

Members of the Wellcome Sanger Institute Tree of Life Management, Samples and Laboratory team are listed here: https://doi.org/10.5281/zenodo.10066175.

Members of Wellcome Sanger Institute Scientific Operations: Sequencing Operations are listed here: https://doi.org/10.5281/zenodo.10043364.

Members of the Wellcome Sanger Institute Tree of Life Core Informatics team are listed here: https://doi.org/10.5281/zenodo.10066637.

Members of the Tree of Life Core Informatics collective are listed here: https://doi.org/10.5281/zenodo.5013541.

Members of the Darwin Tree of Life Consortium are listed here: https://doi.org/10.5281/zenodo.4783558.
