# Supplementary material for: The genome sequence of the crisped pincushion, Ulota crispa (Hedw.) Brid
Source: Wellcome Open Res. 2024 Dec 3;9:711. [Version 1] doi: 10.12688/wellcomeopenres.23429.1 (PMC11803377; doi:10.12688/wellcomeopenres.23429.1)
Supplement: Peer review history

# Reviewer report: Reviewer response for version 1

**DOI:** 10.21956/wellcomeopenres.25839.r115483

**Published:** 2024-12-24

**Authors:** Deniz Aygören Uluer

**Affiliations:** 1 Ahi Evran University, Kirşehir, Turkey

It is a well-written manuscript. I only may suggest to merge the following two sentences:

 It is common and widespread in Europe, and also known from east Asia (China, Russian Far East and Japan, and Taiwan) and western North America, where it is uncommon (Caparrós et al., 2016). It is widespread in Britain and Ireland (Blockeel, 2017).

Are sufficient details of methods and materials provided to allow replication by others?

Yes

Is the rationale for creating the dataset(s) clearly described?

Yes

Are the datasets clearly presented in a useable and accessible format?

Yes

Are the protocols appropriate and is the work technically sound?

Yes

Reviewer Expertise:

Phylogeny, DNA barcoding

I confirm that I have read this submission and believe that I have an appropriate level of expertise to confirm that it is of an acceptable scientific standard.

# Reviewer report: Reviewer response for version 1

**DOI:** 10.21956/wellcomeopenres.25839.r114422

**Published:** 2024-12-17

**Authors:** Kirsten Fisher

**Affiliations:** 1 California State University, Los Angeles, USA

The manuscript describes the methods used to generate a genome sequence for a haploid gametophyte of *Ulota crispa*, an epiphytic moss from the Orthotricaceae. The report is detailed and concise, and conveys the rationale for sequencing the *Ulota crispa *genome in conjunction with the Darwin Tree of Life project. The manuscript also provides some background on the taxonomic history of the *U*. *crispa *species complex, which further supports the potential utility of the focal genome for future studies in this complex.

 The protocols described in the manuscript all appear appropriate, and they are presented in sufficient detail to engender confidence in their technical rigor. Datasets are clearly presented and accessioned, and supporting technical data such as SOPs are also made available in publicly accessible repositories.

 My only suggestion for strengthening the manuscript would be to include a bit more detail with respect to the preparation of the gametophytic tissue that was collected for the PacBio and Hi-C sequencing. The beautiful photograph of the source population depicts a gametophyte cushion that is covered in sporophytes – presumably, all of the sporophytes are the product of self-fertilization since *U. crispa *is monoicous, but the methods only describe two gametophytes being collected. Were collections made at a time to avoid the presence of sporophytes? Or were attempts made to remove the sporophytes from gametophytes prior to tissue preparation? Without this additional detail, I wondered whether the two haplotypes in the sequence data were the product of an out-crossed sporophyte that made its way into the tissue used for DNA extractions or were instead attributable to the two gametophyte cushions that were collected.

Are sufficient details of methods and materials provided to allow replication by others?

Yes

Is the rationale for creating the dataset(s) clearly described?

Yes

Are the datasets clearly presented in a useable and accessible format?

Yes

Are the protocols appropriate and is the work technically sound?

Yes

Reviewer Expertise:

Molecular ecology of bryophytes

I confirm that I have read this submission and believe that I have an appropriate level of expertise to confirm that it is of an acceptable scientific standard.
